# Clinical epigenomics: genome-wide DNA methylation analysis for the diagnosis of Mendelian disorders

**DOI:** 10.1038/s41436-020-01096-4

**Published:** 2021-02-05

**Authors:** Bekim Sadikovic, Michael A. Levy, Jennifer Kerkhof, Erfan Aref-Eshghi, Laila Schenkel, Alan Stuart, Haley McConkey, Peter Henneman, Andrea Venema, Charles E. Schwartz, Roger E. Stevenson, Steven A. Skinner, Barbara R. DuPont, Robin S. Fletcher, Tugce B. Balci, Victoria Mok Siu, Jorge L. Granadillo, Jennefer Masters, Mike Kadour, Michael J. Friez, Mieke M. van Haelst, Marcel M. A. M. Mannens, Raymond J. Louie, Jennifer A. Lee, Matthew L. Tedder, Marielle Alders

**Affiliations:** 1 Molecular Genetics Laboratory, Molecular Diagnostics Division, London Health Sciences Centre, London, ON Canada; 2 Department of Pathology and Laboratory Medicine, Western University, London, ON Canada; 3 Amsterdam University Medical Center, University of Amsterdam, Department of Clinical Genetics, Amsterdam Reproduction and Development Research Institute, Amsterdam, The Netherlands; 4 Greenwood Genetic Center, Greenwood, SC USA; 5 Department of Pediatrics, Division of Medical Genetics, Western University, London, ON Canada; 6 Medical Genetics Program of Southwestern Ontario, London Health Sciences Centre, London, ON Canada; 7 Division of Genetics and Genomic Medicine, Department of Pediatrics, Washington University School of Medicine in St. Louis, St. Louis, MO USA

## Abstract

**Purpose:**

We describe the clinical implementation of genome-wide DNA methylation analysis in rare disorders across the EpiSign diagnostic laboratory network and the assessment of results and clinical impact in the first subjects tested.

**Methods:**

We outline the logistics and data flow between an integrated network of clinical diagnostics laboratories in Europe, the United States, and Canada. We describe the clinical validation of EpiSign using 211 specimens and assess the test performance and diagnostic yield in the first 207 subjects tested involving two patient subgroups: the targeted cohort (subjects with previous ambiguous/inconclusive genetic findings including genetic variants of unknown clinical significance) and the screening cohort (subjects with clinical findings consistent with hereditary neurodevelopmental syndromes and no previous conclusive genetic findings).

**Results:**

Among the 207 subjects tested, 57 (27.6%) were positive for a diagnostic episignature including 48/136 (35.3%) in the targeted cohort and 8/71 (11.3%) in the screening cohort, with 4/207 (1.9%) remaining inconclusive after EpiSign analysis.

**Conclusion:**

This study describes the implementation of diagnostic clinical genomic DNA methylation testing in patients with rare disorders. It provides strong evidence of clinical utility of EpiSign analysis, including the ability to provide conclusive findings in the majority of subjects tested.

## INTRODUCTION

Mendelian disorders are estimated to occur at a rate of 40 to 82 per 1,000 live births.^1^ However, if all congenital anomalies are considered, approximately 8% of individuals are estimated to have a genetic disorder before reaching adulthood.^2^ Clinical presentations in most genetic disorders include developmental delay and intellectual disability (DD/ID), sometimes in combination with other features including dysmorphism, neuromuscular phenotypes, and other systemic constellations of syndromes.^3^ Despite rapid advances in our understanding of the human genome, nearly two-thirds of the patients with suspected rare genetic disorders remain without a conclusive molecular genetic diagnosis.^4^

Evolution of genetic testing from single-nucleotide assessment to clinical exome and genome sequencing, while increasing the diagnostic yield to an average of 36%,^4^ has also resulted in a significant increase in ambiguous or uncertain genetic findings, referred to as variants of unknown clinical significance (VUS). Despite concerted efforts to standardize guidelines for the interpretation of sequence variants^5^ and to define the functional evidence for variant classification,^6^ a large proportion of VUS remain without conclusive clinical interpretation. Also, the understanding of the impact of genetic variation outside of protein-coding DNA sequences is very limited, and as such, the majority of genetic testing in clinical laboratories is focused on exonic and short surrounding intronic sequences. Family variant cosegregation studies, in silico prediction algorithms, and gene-specific functional studies may help resolve VUS findings, but in the majority of cases these are not available, feasible, or conclusive.

One functional consequence of genetic defects in patients with hereditary neurodevelopmental disorders is the disruption of genomic DNA methylation.^7^ DNA methylation is an epigenetic modification, resulting in changes in structural and chemical properties of the DNA, impacting molecular mechanisms including chromatin assembly and gene transcription.^8^ Our group and others have demonstrated that individuals among a growing number of rare disorders exhibit DNA methylation “episignatures” or “EpiSigns” as highly sensitive and specific DNA methylation biomarkers.^9–22^ These genome-wide DNA methylation profiles currently include over 40 rare disorders in association with more than 60 genes, and can be gene domain, gene level, as well as protein complex specific. DNA methylation episignatures are detectable in peripheral blood and are highly sensitive and specific for each disorder. As such, they represent effective biomarkers for the testing of patients with a broadening range of neurodevelopmental genetic conditions, as well as a reflex functional test for patients with ambiguous genetic test findings or clinical phenotypes.^23^

Genomic DNA methylation analysis is also adaptable to the routine analytical processes in clinical laboratories. Cytosine methylation is a highly stable analyte, and genome-wide DNA methylation data can be generated on a microarray platform. In parallel with episignature screening, genomic DNA methylation analysis enables concurrent and highly sensitive and specific assessment of imprinting disorders^24^ and fragile X syndrome,^25^ enabling further test consolidation in this patient population. One key technical challenge in the clinical setting is data analysis. This requires the development of large-scale reference DNA methylation databases, including disorder and tissue-specific reference data sets and controls, and sophisticated analytical processes including machine learning algorithms as analytical classifiers. Testing must be performed in a regulated clinically certified environment, with adherence to the required quality management procedures, and clinical quality metrics, all under professional clinical oversight.

In this study we describe the implementation and validation of EpiSign, a clinical genome-wide DNA methylation test for patients with rare Mendelian disorders, based on the Illumina Infinium methylation array technology and the EpiSign Knowledge Database (EKD). We describe the quality metrics and clinical validation metrics within an integrated network of licensed academic nonprofit clinical laboratories in Europe, Canada, and the United States. We describe the clinical performance and the diagnostic yield in subjects tested between initiation of the service in November 2019 to June 2020. This study demonstrates the clinical utility of genomic DNA methylation testing in patients with Mendelian neurodevelopmental disorders.

## MATERIALS AND METHODS

### Clinical validation and patient cohorts

The validation cohort (Table S1) was designed to clinically validate and assess quality metrics of the EpiSign test across the EpiSign diagnostic laboratory network, and consisted of 211 archived peripheral blood DNA samples including samples with confirmed diagnosis of one of 43 genetic syndromes included in the EpiSign v2 genome-wide DNA methylation assay (Table 1), or controls. The genetic variation in these specimens were classified as pathogenic or likely pathogenic based on the American College of Medical Genetics and Genomics (ACMG) guidelines for interpretation of genomic sequence variants.^5^ Technical inter and intrarun replicates were assessed for concordance based on methylation variant pathogenicity (MVP) score (within 0.05) and clustering analysis. The clinical testing cohort (Table S2) consists of peripheral blood DNA samples from 207 subjects, referred by physicians based on individual clinical discretion, who have received clinical EpiSign testing. All subjects provided informed consent for clinical genetic testing as part of pretest counseling.

**Table 1:** Disorders detectable by EpiSign v2.

Disease/disorder	Causative gene(s)/region	New in EpiSign V2	Validation cohort positives	Clinical cohort positives
ɑ-thalassemia/mental retardation X-linked syndrome (ATR-X)	*ATRX* (301404)	No	2	4
Autism, susceptibility to, 18 (AUT18)	*CHD8* (610528)	Yes	6	3
BAFopathies: Coffin–Siris (CSS1–4, and 8) and Nicolaides–Baraitser (NCBRS) syndromes	*ARID1B* (135900), *ARID1A* (614607)*, SMARCB1* (614608), *SMARCA4* (614609), *SMARCC2* (618362), *SMARCA2* (601358)	*ARID1A*, *SMARCC2*	28	10
Blepharophimosis intellectual disability syndrome, SMARCA2 type	*SMARCA2* (OMIM not available, PMID: 32694869)	Yes	2	
Börjeson–Forssman–Lehmann syndrome (BFLS)	*PHF6* (301900)	Yes	1	
Cerebellar ataxia, deafness, and narcolepsy, autosomal dominant (ADCADN)	*DNMT1* (604121)	No		
CHARGE syndrome	*CHD7* (214800)	No	6	4
Cornelia de Lange syndrome (CdLS)	*NIPBL* (122470), *RAD21* (614701), *SMC3* (610759), *SMC1A* (300590)	No	11	6
Down syndrome	Trisomy 21 (190685)	No	1	
Epileptic encephalopathy, childhood-onset (EEOC)	*CHD2* (615369)	Yes	1	
Floating Harbor syndrome (FLHS)	*SRCAP* (136140)	No		
Genitopatellar syndrome (GTPTS) and Ohdo syndrome, SBBYSS variant (SBBYSS)	*KAT6B* (606170; 603736)	No	3	2
Helsmoortel–van der Aa syndrome (HVDAS)^a^	*ADNP* (615873)	No	9	2
Hunter–McAlpine syndrome (HMA)	Chr5q35-qter duplication (601379)	Yes		
Immunodeficiency–centromeric instability–facial anomalies syndrome (ICF)^b^	*DNMT3B* (242860), *CDCA7* (616910), *ZBTB24* (614069), *HELLS* (616911)	Yes		
Kabuki syndrome	*KMT2D* (147920), *KDM6A* (300867)	*KDM6A*	12	5
Kleefstra syndrome	*EHMT1* (610253)	Yes	2	2
Koolen–de Vries syndrome (KDVS)	*KANSL1* (610443)	Yes	5	
Mental retardation, autosomal dominant 23 (MRD23)	*SETD5* (615761)	Yes	4	
Mental retardation, autosomal dominant 51 (MRD51)	*KMT5B* (617788)	Yes		
Mental retardation, X-linked 93 (MRD93)	*BRWD3* (300659)	Yes	1	1
Mental retardation, X-linked 97 (MRD97)	*ZNF711* (300803)	Yes		1
Mental retardation, X-linked syndromic, Nascimento-type (MRXSN)	*UBE2A* (300860)	Yes	1	
Mental retardation, X-linked, Snyder–Robinson type (MRXSSR)	*SMS* (309583)	Yes		1
Mental retardation, X-linked, syndromic, Claes–Jensen syndrome (MRXSCJ)	*KDM5C* (300534)	No	6	1
PCR2 complex (Weaver (WVS) and Cohen–Gibson (COGIS)	*EZH2* (277590), *EED* (617561)	Yes		
Rahman syndrome (RMNS)	*HIST1H1E* (617537)	Yes		
Rubinstein–Taybi syndrome (RSTS)	*CREBBP* (180849)*, EP300* (613684)	Yes	2	3
SETD1B-related syndrome	*SETD1B* (619000)	Yes		1
Sotos syndrome	*NSD1* (117500)	No	8	4
Tatton–Brown–Rahman syndrome (TBRS)	*DNMT3A* (615879)	Yes	1	1
Wiedemann–Steiner syndrome (WDSTS)	*KMT2A* (605130)	Yes	3	2
Williams–Beuren deletion syndrome (WBS) and Williams–Beuren regions duplication syndrome (Dup7)^c^	7q11.23 deletion (194050)/duplication (609757)	No	2	
Wolf–Hirschhorn syndrome (WHS)	Chr4p16.13 deletion (194190)	Yes		
Fragile X syndrome (FXS)	TNR/*FMR1* (300624)	No	6	
Mental retardation, FRA12A type	TNR/*DIP2B* (136630)	No		1
Angelman syndrome (AS)	ID/*UBE3A* (105830)	No	6	1
Prader–Willi syndrome	ID/15q11 (*SNRPN*, *NDN*) (176270)	No	3	
Silver–Russell syndrome 1 (SRS1)	ID/11p15.5 (180860)	No	5	1
Beckwith–Wiedemann syndrome (BWS)	ID/11p15 (*ICR1*, *KCNQ1OT1*, *CDKN1C*) (130650)	No	4	
Silver–Russell syndrome 2 (SRS2)	ID/7p11.2 (180860)	No		
Temple syndrome	ID/14q32 (616222)	No		
Kagami–Ogatta syndrome (KOS)	ID/14q32 (608149)	No	2	

OMIM number listed in parentheses adjacent to disorder name. The following list of genes have been classified as having reduced sensitivity and more moderate signatures based on signature strength, limited reference cohort size, or types of variants that have been tested: *CHD8*, *PHF6*, *DNMT3B*, *CDCA7*, *ZBTB24*, *HELLS*, *SETD5*, *KMT5B*, *BRWD3*, *ZNF711*, *KAT6B*, *SMS*, *DNMT3A*.

*ID* imprinting disorder, *TNR* trinucleotide repeat disorder.

^a^*ADNP* has two distinct signatures depending on where in the gene the variant occurs. HVDAS_T signature includes variants that occupy the N- and C-terminus of the gene and HVDAS_C includes variants in the central region of the gene including the nuclear localization signal of the protein.

^b^ICF1 exhibits one signature while ICF 2, 3, and 4 exhibit a separate, common signature.

^c^These two deletion/duplication syndromes exhibit symmetrical increased/decreased DNA methylation signatures, respectively.

DNA methylation analysis was performed using the Illumina Infinium EPIC bead chip arrays as previously described^9^ by the clinical testing laboratories: Greenwood Genetic Center (Greenwood, SC, USA) and Amsterdam University Medical Center and partner labs (Amsterdam, Netherlands). Data from validation and clinical testing specimens (November 2019 and June 2020) were blinded and submitted to the clinical bioinformatics laboratory (Molecular Diagnostics Laboratory, London Health Sciences Centre, Western University, London, Canada) through a secure file transfer protocol and housed on the hospital clinical encrypted servers.

### DNA methylation data analysis

Analysis of the DNA methylation array data was performed by the clinical bioinformatics laboratory using Illumina Infinium EPIC arrays. Methylation data for each sample were compared to the established DNA methylation episignatures for the 43 disorders (Table 1) which are part of the EpiSign clinical test. EpiSign analysis utilized the EKD, a clinical database with >5,000 peripheral blood DNA methylation profiles including disorder-specific reference cohorts and normal (general population samples with various age and racial backgrounds) controls housed at London Health Sciences Centre Molecular Diagnostics Laboratory (https://www.lhsc.on.ca/palm/molecular.html). Individual DNA methylation data for each subject were compared with the EKD using the support vector machine (SVM) based classification algorithm for EpiSign disorders. Methylation variant Pathogenicity (MVP) score is generated ranging between 0 and 1, representing the confidence of prediction for the specific class the SVM was trained to detect. Conversion of SVM decision values to these scores was carried out according to the Platt scaling method.^26^ Classification for a specific EpiSign disorder included MVP score assessment with a general threshold of >0.5 for positive, <0.1 negative, 0.1–0.5 inconclusive or low confidence, hierarchical clustering and the multidimensional scaling (MDS) of subject’s methylation data relative to the disorder-specific EpiSign probe sets and controls. A detailed description of this analytics protocol was described previously.^9,27^

### Clinical assessment and reporting

DNA methylation analysis results were clinically verified by a board-certified clinical molecular geneticist at the clinical bioinformatics laboratory. Result categories include positive (matching an EpiSign disorder), negative (not matching any EpiSign disorder), and inconclusive (described in detail in results). The report is then reviewed and verified by a board-certified laboratory professional in the clinical testing laboratory, and a clinical report describing the EpiSign results (positive, negative, or inconclusive for a particular episignature) is issued to the requesting physician.

## RESULTS

### EpiSign clinical validation

The 211 validation specimens are described in Table S1. Data were generated at the clinical testing laboratories, anonymized, and submitted to the clinical bioinformatics laboratory for EpiSign analysis. EpiSign analysis was concordant with the previous genetic findings in 207/211 samples. A positive control cohort included 143 samples with various genetic disorders with previously reported DNA episignatures, imprinting and uniparental disomy disorders, and fragile X syndrome. Of these, 139 were concordant for the expected episignature. The discordant cases included a subject (Val118) with a previously reported likely pathogenic variant, *KANSL1* (NM_001193466.1): c.297_307del; p.Gly100Glnfs*6, related to Koolen–de Vries syndrome (KDVS), and another subject (Val26) with a previously reported likely pathogenic variant, *CREBBP* (NM_004380.2):c.4480C>A; p.Pro1494Thr, related to the Rubinstein–Taybi syndrome (RSTS). In the case Val118 with the *KANSL1* likely pathogenic variant, and clinical features consistent with KDVS, the MVP score for KDVS was zero. Some individuals carry a duplication harboring part of the *KANSL1* gene elsewhere in the genome, which may cause variants in the duplicated region to be erroneously assigned to the *KANSL1* gene. However, assessment of exome sequencing data, array comparative genomic hybridization (array CGH), and multiplex ligation-dependent probe amplification (MLPA) analysis showed no evidence of this this duplication. The MVP score in the second case, Val26, with *CREBBP*(NM_004380.2):c.4480C>A, p.(Pro1494Thr), for RSTS was zero. Potential mosaicism of the *CREBBP* variant was not apparent. The remaining two discordant cases (Val128 and Val140) both had previously identified *SMARCC2* pathogenic variants. Fifty-six samples were normal reference controls (NC) and were all concordant. The remaining 12 samples (other controls in Table S1) included samples with previous genetic findings that are currently undetectable by EpiSign analysis including samples with low-level mosaicism for imprinting disorders, fragile X female heterozygotes, 16p11del and *ARID2* pathogenic variant. There were 10 interrun duplicates (samples processed in different array batches) and 18 intrarun duplicates (replicate samples processed in the same batch), and all were concordant. EpiSign v2 validation included 55 replicates of samples used in v1 validation and all were concordant.

### EpiSign clinical testing

EpiSign analysis includes genetic conditions that exhibit DNA methylation episignatures as well as the common imprinting disorders and fragile X (Table 1). A total of 207 subjects were tested by EpiSign analysis and reported in the period between September 2019 and June 2020. Of these, 57 specimens were positive for an episignature, 146 were negative, and 4 were inconclusive (Table S3). Of the 207 subjects, a total of 136 patient samples had a previous VUS finding, and of these, 48 (35.3%) had DNA methylation profiles positive for one of the EpiSigns. Most of the positive cases had robust DNA methylation profiles (MVP scores >0.9 with unambiguous MDS and clustering analysis) with some positive cases showing moderate, but positive profiles (reduced but positive MVP score, or closer to borderline MDS clustering). Eighty-six cases had no evidence of a DNA methylation episignature. The remaining two VUS cases were inconclusive.

**Fig. 1:**
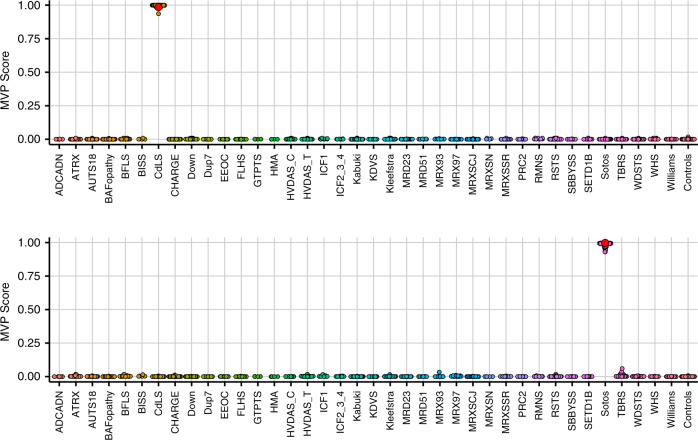
A multiclass supervised classification system. This classification system, referred to as a methylation variant pathogenicity (MVP) score, can classify samples using the 43 episignatures in EpiSign v2. Shown here are examples using the Cornelia de Lange syndrome (CdLS) signature (top) and Sotos signature (bottom), applied to over 1,500 samples from subjects with various neurodevelopmental syndromes or from healthy controls. In each case, samples from the respective syndrome all have high scores while samples from other syndromes and controls all have low scores, demonstrating the sensitivity and specificity of the classifier. The likely pathogenic variants described in Fig. 2 (CdLS) and 3 (Sotos) are shown here larger and in red.

**Fig. 2:**
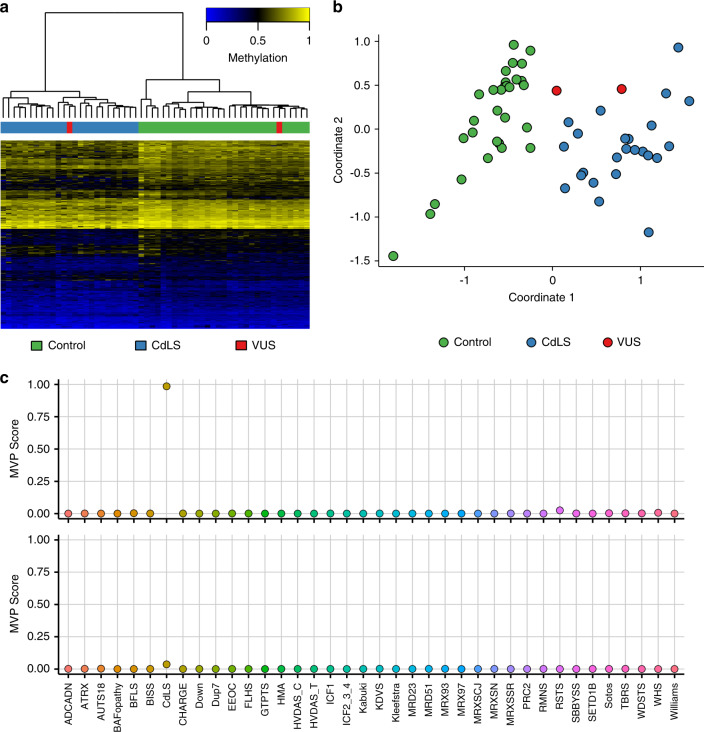
DNA methylation (EpiSign) analysis of peripheral blood from two subjects with variants of unknown clinical significance (VUS) in *SMC1A*, the causative gene for Cornelia de Lange syndrome (CdLS) type 2. The variants are *SMC1A*:c.598A>C, p.(Lys200Gln) (labeled red and clustering with *CdLS* samples) and *SMC1A*:c.1280A>G, p.(Glu427Gly) (labeled red and clustering with control samples). (**a**) Hierarchical clustering and (**b**) multidimensional scaling plot of subjects with a confirmed CdLS episignature, controls, and the VUS under investigation. The *SMC1A*:c.598A>C VUS clustering with the CdLS samples indicates it has a DNA methylation signature similar to that seen in other CdLS samples and suggesting that the variant is pathogenic, while *SMC1A*:c.1280A>G is likely benign. (**c**) Methylation variant pathogenicity (MVP) score, a multiclass supervised classification system capable of discerning between the 43 different episignatures in EpiSign V2, was applied to the *SMC1A*:c.598A>C likely pathogenic variant (top) and the *SMC1A*:c.1280A>G likely benign variant (bottom). This classification system generates a probability score for each episignature, with a score near 1 indicating that the sample has an episignature similar to the reference episignature.

**Fig. 3:**
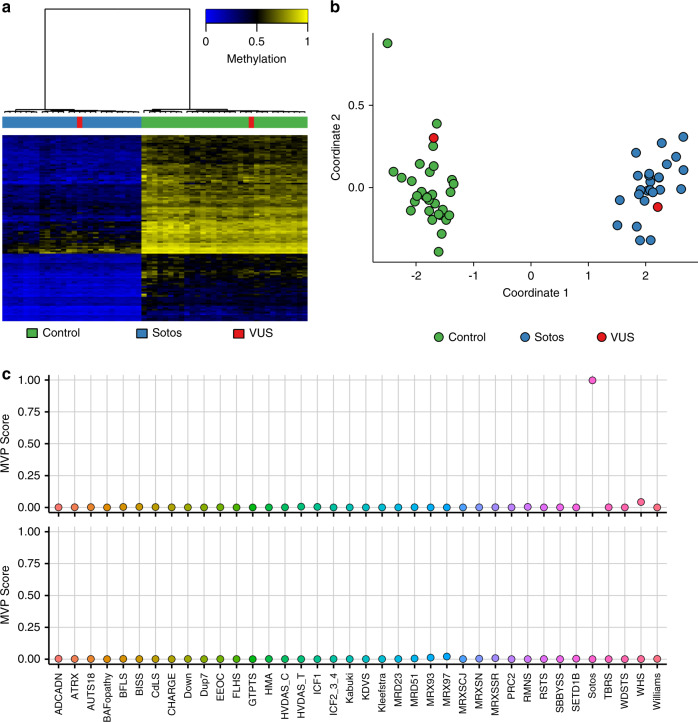
DNA methylation (EpiSign) analysis of peripheral blood from two subjects with variants of unknown clinical significance (VUS) in *NSD1*, the causative gene for Sotos syndrome type 1. The variants are *NSD1*:c.4982G>C,p.Cys1661Ser) (labeled red and clustering with *NSD1* samples) and *NSD1*:c.3331G>T,p.Asp1111Tyr (labeled red and clustering with control samples). (**a**) Hierarchical clustering and (**b**) multidimensional scaling plot of subjects with a confirmed Sotos episignature, controls, and the VUS under investigation. The *NSD1:*c.4982G>C VUS clustering with the Sotos samples indicates it has a DNA methylation signature similar to that seen in other Sotos samples and suggesting that the variant is pathogenic, while *NSD1:*c.3331G>T is likely benign. (**c**) Methylation variant pathogenicity (MVP) score, a multiclass supervised classification system capable of discerning between the 43 different episignatures in EpiSign v2, was applied to the *NSD1:*c.4982G>C likely pathogenic variant (top) and the *NSD1:*c.3331G>T likely benign variant (bottom). This classification system generates a probability score for each episignature, with a score near 1 indicating that the sample has an episignature similar to the reference episignature.

Figure 1 shows examples of MVP score plots for Cornelia de Lange and Sotos syndromes. Figure S1 shows the representative genomic loci along with reference control and positive DNA methylation profiles for imprinting and fragile X disorders listed in Table 1. For specimens that screen positive for a specific MVP score, hierarchical clustering and multidimensional scaling are performed. Figure 2 shows data for two subjects with VUS in *SMC1A*, the causative gene for Cornelia de Lange syndrome (CdLS) type 2, *SMC1A*:c.598A>C, p.(Lys200Gln) and *SMC1A*:c.1280A>G, p.(Glu427Gly). The *SMC1A*:c.598A>C, p.(Lys200Gln) variant was reclassified as likely pathogenic, ACMG category 2, and the *SMC1A*:c.1280A>G, p.(Glu427Gly) variant did not show evidence of pathogenicity based on the current reference episignatures. Other examples depicted in Fig. 3 show data from two subjects with VUS in *NSD1*, the causative gene for Sotos syndrome type 1, *NSD1*:c.4982G>C, p.(Cys1661Ser) and *NSD1*:c.3331G>T, p.Asp1111Tyr. The *NSD1*:c.4982G>C, p.(Cys1661Ser) was reclassified to likely pathogenic, ACMG category 2, while *NSD1*:c.3331G>T, p.Asp1111Tyr, which clusters with controls, showed no evidence of pathogenicity. Similar analysis is performed for all subjects tested with findings summarized in Table S3.

One of the four inconclusive clinical testing cases, Clin77, had a *ARID2*:c.988_1008del, p.(Leu330_Gly336del) VUS. MVP score for BAFopathy using the EpiSign v1 was slightly elevated (0.2) but below the established 0.5 cutoff and above the 0.1 cutoff for reference normal controls. The updated EpiSign v2 reanalysis showed no evidence of the elevated BAFopathy score; however, MRD23 score remained elevated. MDS profile showed clustering between BAFopathy cohort and controls (Figure S2). The current BAFopathy episignature is trained on positive cases with pathogenic variants in *ARID1B*, *ARID1A*, *SMARCB1*, *SMARCA2*, and *SMARCA4*. Since BAF complex–associated *ARID2* positive references are not represented, it was not possible to confidently rule in/out a BAFopathy episignature. Case Clin203 had a previously identified *ADNP*:c.46C>G, p.(Arg16Gly) VUS. The *ADNP* gene has two distinct EpiSigns^11^ as a result of truncating variants in two distinct protein domains; the 5’ being defined by the variants ranging from c.56 to c.1287. The MVP score for this subject was 0.03, which is within the normal range, but MVP scores for all other conditions were zero. Due to the strong hypomethylation observed with this episignature, MDS analysis clearly separates the reference from the positive cohort, and this sample plots between the two (Figure S2). In lieu of these findings and because the variant lies outside of the established EpiSign domain, the result was reported as inconclusive. For case Clin120, the MVP scores were within the expected reference range but showed slight elevation for MRX97 (0.02) (Figure S2). Although this value is within reference control limits, the currently defined episignature for MRX97 is mild and derived from a limited positive reference cohort. Since this subject’s phenotype had a partial overlap with MRX97 the result was reported as inconclusive. The final case, Clin202, was referred because of clinical features consistent with BAFopathy disorder and no variants identified in BAF complex genes. This sample clustered between BAFopathy and the control samples by the MDS analysis (Figure S2). Although the MVP score was within the normal reference range for BAFopathy and all other EpiSign disorders, as in case Clin77, and we could not rule out involvement of other yet unmapped BAF complex genes.

### Notable clinical cases

**Fig. 4:**
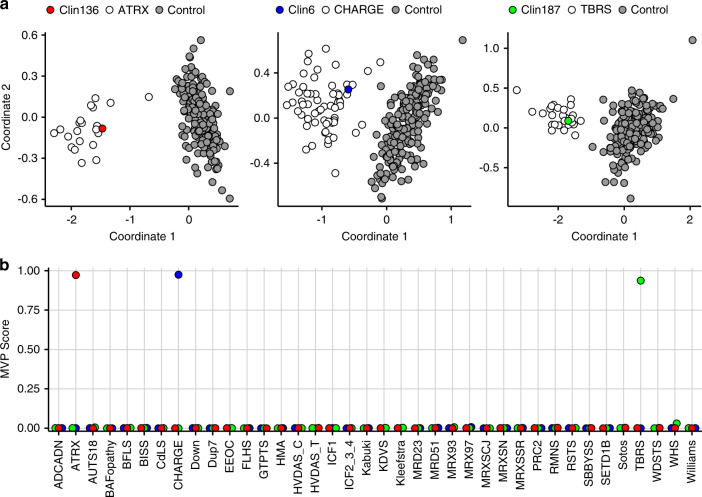
Clinical case EpiSign assignment. Assessment of the notable clinical cases. (**a**) Principal component analysis (PCA) plots for Clin136 (no genetic variant identified), Clin6 (no genetic variant identified), and Clin187 (*DNMT3A*:c.2146G>A [p.Val716Ile]). (**b**) Corresponding methylation variant pathogenicity (MVP) scores for the notable clinical cases. Both PCA plots and MVP scores provide conclusive evidence for EpiSign classification of these clinical cases.

While one use of EpiSign is to help resolve VUS, there are scenarios where a DNA methylation episignature is the only molecular diagnostic finding. Case Clin136 was referred for EpiSign analysis due to clinical features consistent with ATRX-related syndrome. However, previous genetic testing of this individual did not identify any alterations in the *ATRX*, using targeted and exome sequencing. In contrast, EpiSign analysis conclusively confirmed presence of a specific *ATRX*-specific DNA methylation signature (Fig. 4). Hence, for this subject, DNA methylation profiling remains the only molecular diagnosis explaining their clinical presentation.

A similar scenario with a different outcome involves subject Clin6 who was referred for EpiSign analysis as a result of negative molecular sequencing (*CHD7* and *SEMA3E*), negative microarray, and negative exome analysis with the phenotype strongly suggestive of CHARGE syndrome. EpiSign analysis identified a DNA methylation profile specific for *CHD7*, consistent with the clinical diagnosis (Fig. 4). As a result, follow-up molecular studies identified the causative deep intronic variant in *CHD7* and confirmed the molecular diagnosis. Details of this case resulting from extensive genomic evaluation by the Undiagnosed Diseases Network (https://undiagnosed.hms.harvard.edu/) are described in a separate manuscript in preparation. Hence, EpiSign analysis provided the necessary evidence for a more in-depth and focused molecular analysis, ultimately leading to the definitive molecular genetic diagnosis.

Case Clin187 highlights an example where extensive molecular genetic testing was performed without a molecular diagnosis, referred to as a diagnostic odyssey. Findings included exome sequencing with *PTCHD1*:c.605G>A (p.Arg202Gln) VUS, maternal; *VPS13B*:c.1520A>G (p.Asn507Ser) VUS, paternal; *LAMC3*:c.4415G>A (p.Arg1472Gln), maternal; *EZH2*:c.2110+6T>G VUS, maternal; and normal findings for: *FMR1*, *MECP2*, SNP 6.0 microarray, *NSD1* (sequencing and MLPA), extensive biochemical workup, and X-inactivation studies. However, EpiSign analysis showed an episignature associated with *DNMT3A*, the gene involved in Tatton–Brown–Rahman syndrome (Fig. 4). Follow up sequencing of the *DNMT3A* gene revealed a missense likely pathogenic variant *DNMT3A*:c.2146G>A (p.Val716Ile). As an infant, the patient met motor and speech milestones but regressed at 18 months, losing speech, attentiveness, and responsiveness to directions. Now at age 17 years, she has overgrowth (all growth parameters greater than the 97th centile), limited speech, echolalia, mild intellectual disability, and autism spectrum disorder.

## DISCUSSION

This paper describes the implementation of genome-wide DNA methylation analysis in clinical testing of individuals with rare genetic disorders. It represents a major milestone in molecular diagnostics as it advances clinical genetic testing beyond assessment of the DNA nucleotide sequence, or genomics, to clinical epigenomics. While targeted molecular assays for assessment of DNA methylation defects of imprinting disorders^28,29^ and fragile X syndrome^30^ have been around for decades, EpiSign enables simultaneous assessment for these, as well as a rapidly expanding number of genetic neurodevelopmental disorders exhibiting DNA methylation episignatures.

### Clinical impact

The primary clinical utility of EpiSign analysis is the assessment and reclassification of VUS in genes with existing episignatures,^15^ and the assessment of genetically unsolved individuals with suspected hereditary conditions.^14^ While this clinical utility has been described in a research setting in targeted patient cohorts, this study focuses on the assessment of the impact of genome-wide DNA methylation analysis in a clinical setting in a prospective unselected patient population. The majority of the subjects tested in the clinical cohort (136; 65.7%) had previously identified genetic VUS, and of these, 134 (98.5%) received a conclusive EpiSign report (positive or negative), of which 48 (35.3%) showed a distinct EpiSign. For the 86 (63.2%) VUS cases with negative EpiSign results, lack of an episignature is considered strong evidence for rule out of these disorders. However, due to the possibility of the existence of additional/alternate yet unmapped episignatures in these genes, as has been demonstrated in *ADNP*^11^ and *SMARCA2*,^31^ we currently stop short of fully ruling out pathogenicity. Only 2 (1.5%) VUS cases remained unclassified. These findings represent a significant advancement in clinical variant assessment over currently available methodologies. While alternative approaches for classification of VUS exist, including functional and family segregation studies, these are not always available, feasible, or conclusive.^32^ EpiSign analysis is less susceptible to those limitations as it assesses the same tissue (patient’s peripheral DNA) used for sequence analysis, and it generally does not require assessment of other family members. From a patient, family, and clinical management perspective, providing the patient and care team with a rapid diagnosis relating to the functional impact of the genetic condition, which for a microarray-based test can be achieved within 1–2 weeks, may be most beneficial to decision-making.

Of the remaining 71 subjects without the previously identified genetic findings, 8 subjects (11.3%) had a positive EpiSign result, demonstrating evidence for the clinical utility of EpiSign analysis in a broader patient population. Given that these subjects already had varying degrees of targeted genetic investigations completed with no conclusive findings, some of the EpiSign disorders had effectively been ruled out already. These findings taken together suggest there may be a health systems value proposition of instigating use of EpiSign earlier in the diagnostic journey of individuals with rare disorders.

Application of this technology to the broader patient populations will depend on the rate of discovery of gene and disorder-specific episignatures. As a corollary, implementation of chromosomal microarrays as a first-tier diagnostic test was primarily contingent upon increased diagnostic yield compared with karyotyping (from 5% to 10–15%) resulting from years of research and discovery of novel microdeletion and duplication syndromes, often involving large clinical databases and registries.^33,34^ Similarly, there are now major efforts underway to assess the clinical utility and the health systems impact, and to accelerate the rate of episignature discovery including a national-scale trial (“Beyond Genomics: Assessing the Improvement in Diagnosis of Rare Diseases using Clinical Epigenomics in Canada [EpiSign-CAN]”), which will compare the impact of DNA methylation analysis as a first-line versus a second-line test in 4,000 individuals with suspected rare disorders while assessing EpiSigns in 100 additional genetic conditions (https://www.genomecanada.ca/en/beyond-genomics-assessing-improvement-diagnosis-rare-diseases-using-clinical-epigenomics-canada).

The clinical cases presented in detail highlight some important implications of this technology. One is the sequential use of EpiSign analysis with genetic testing. While using EpiSign to investigate individuals with VUS or related clinical presentations provides demonstrated value, EpiSign can also uncover genetic disorders that were not initially suspected. We have previously demonstrated an incremental diagnostic yield of approximately 3% in patients with prior extensive genomic testing but without a genetic diagnosis.^9,14^ Existence of a specific DNA methylation pattern can guide the molecular assessment, and in some cases resolve complex diagnostic odysseys, which can have a huge impact on patient care and the related health systems costs.^35,36^

### Clinical service delivery

EpiSign testing is performed using an integrated model involving primary labs performing and reporting test results with informatics and databasing centralized in the tertiary clinical bioinformatics laboratory, similar to the field of noninvasive prenatal testing for aneuploidy.^37^ The key benefits of this model involve standardization and coordinated quality management and quality assessment procedures, ensuring consistency across the different provider laboratories, which is critical given the inherent nature and complexity of this analysis. This also allows for more rapid expansion of reference DNA methylation databases and facilitates continuous optimization of the underpinning analytical algorithms. As the EKD expands, the reference machine learning–derived algorithms that form the basis of individual EpiSigns become more sensitive and specific,^9^ enabling regular and documented updates to the analytical software, with the appropriate quality metrics and quality control documentation and version controls.

### Limitations

There are a number of challenges related to introducing a diagnostic modality to the clinical laboratory, and EpiSign is not an exception. Unlike DNA sequencing, DNA methylation analysis is limited to peripheral blood where large reference databases are available. Other factors including age, sex, and environmental exposures can also impact the analysis and need to be accounted for in analytical processes.^38,39^

DNA methylation episignatures can be susceptible to technical variation such as sample processing data batch effects, as well as biological parameters such as mosaicism. The validation cohort included a number of low-level mosaic imprinting disorders that may not be readily detectable by the EpiSign assay. In our previous work we were able to detect mosaicism in imprinting disorders^24^ and fragile X,^25^ at levels >20%, but due to normal control variability (Figure S1), this is currently not routinely possible for samples with lower-level mosaicism. Mosaicism is also a limitation for detection of other EpiSigns, and may provide an explanation for some of the discordant samples. We have previously demonstrated that total gene dosage dilutes the intensity of the EpiSign signal, as in heterozygous females in the X-linked *KDM5C*-related Claes–Jensen syndrome;^16^ however, here, as a result of having a reference cohort we were able to derive a specific and sensitive MVP score for heterozygous females. In addition to mosaicism, a possible reason for nonconcordance in a laboratory setting could be sample mix up. Alternatively, discordance of sample Val26 with *CREBBP*:c.4480C>A, p.(Pro1494Thr) may be explained by the variant not actually being pathogenic, or an existence of a yet unmapped episignature in this gene.

Other biologically based limitations of EpiSign analysis can be highlighted by the four inconclusive samples from the clinical testing cohort. The current BAFopathy episignature is trained on positive cases with pathogenic variants in *ARID1B*, *ARID1A*, *SMARCB1*, *SMARCA2*, and *SMARCA4*.^17^ Hence, it is not possible to completely rule out pathogenicity of a variant of another BAF complex gene as for the case with the *ARID2* variant. An alternative scenario is highlighted by *ADNP*, which was the first gene in which the existence of multiple, domain-specific episignatures was described.^11^ Although majority of the EpiSign genes currently have a single common episignature mapped, assessment of pathogenicity of variants outside the established reference range and variant type warrants caution, as in the inconclusive case Clin203.

An overarching challenge with this technology is the rarity of Mendelian disorders. While the population prevalence of rare diseases is 3.5–5.9%, equating to 263–446 million persons affected globally, given that this number encompasses >5,000 diseases, the prevalence of rare disorders ranges between 1–5 per 10,000 and <1/1,000, 000.^40^ Generation of EpiSigns requires cohorts of subjects with gene-specific pathogenic variants, which is currently possible for the more prevalent disorders. Also, as many of the episignatures are mild in scale, the size of the reference cohort is directly correlated to the level of sensitivity of the assay. Hence, occasionally, as in the case Clin120 for example, the results may be inconclusive.

While the recommendations for application of the functional evidence in genetic testing now exist,^6^ there are currently no specific guidelines for the clinical interpretation of genomic DNA methylation findings. EpiSign employs the use of DNA methylation data as a surrogate for evidence of the underpinning genetic defects that may or may not be detectable using the current molecular testing modalities. Another challenge is that our current knowledge of the full scope of these genetically associated epiphenotypes is limited. Hence, while on the one hand a confirmation of a related episignature in a patient with a genetic VUS may be considered a molecular diagnosis, a negative result in a patient with or without a known genetic variant is not an absolute rule-out and the analysis would be considered a molecular screen. A more complex challenge is a confirmation of an episignature in absence of a detectable genetic variation, in particular when it is observed in a patient with a matching clinical diagnosis. In this case EpiSign is the standalone molecular diagnosis.

### Conclusion

This study describes the implementation of clinical genomic DNA methylation testing in patients with rare disorders. It demonstrates strong evidence of clinical utility, including the ability to provide conclusive diagnoses in a significant proportion of subjects tested. It also highlights the limitations and challenges with implementation and use of this diagnostic modality. As this technology evolves, the number and type of rare disorders with EpiSigns is going to expand, increasing its clinical utility. While the current clinical use of EpiSign focuses on cases with VUS, and very specific clinical presentations, expanding clinical utility of this test may justify its application earlier in the diagnostic journey, in a broader patient population. Larger-scale studies, such as EpiSign-CAN, are necessary to assess the diagnostic yield and health system impact as either a first-line test or in unresolved cases post–genomic assessment. Finally, the development of clinical guidelines for use and application of clinical epigenomic technologies is warranted.

## Supplementary information

Supplementary Figures

Table S1

Table S2

## Data Availability

The summarized, anonymized data for each subject are described in the study. The raw anonymized DNA methylation data are available from the authors upon request. Software used in this study is publicly available and detailed analytical methodology is as previously reported.^9^
