# Supplementary material for: Clinical epigenomics: genome-wide DNA methylation analysis for the diagnosis of Mendelian disorders
Source: Genet Med. 2021 Feb 5;23(6):1065–74. doi: 10.1038/s41436-020-01096-4 (PMC8187150; doi:10.1038/s41436-020-01096-4)
Supplement: Supplementary file 1 — Supplementary Figures [file 41436_2020_1096_MOESM1_ESM.pdf]

Figure S1

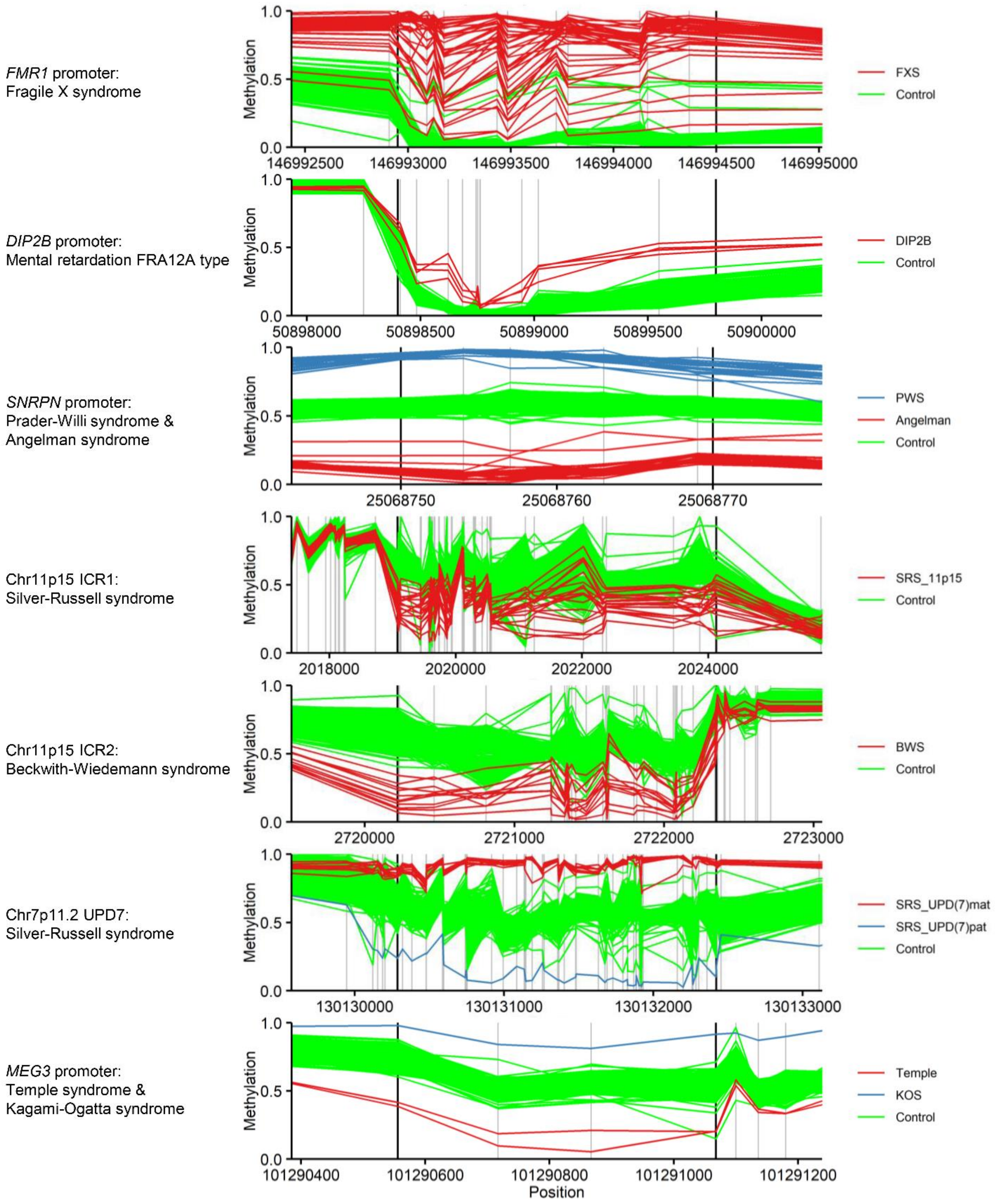
FMR1 promoter:
Fragile X syndrome
DIP2B promoter:
Mental retardation FRA12A type
SNRPN promoter:
Prader-Willi syndrome &
Angelman syndrome
Chr11p15 ICR1:
Silver-Russell syndrome
Chr11p15 ICR2:
Beckwith-Wiedemann syndrome
Chr7p11.2 UPD7:
Silver-Russell syndrome
MEG3 promoter:
Temple syndrome &
Kagami-Ogatta syndrome
Methylation
Position
FXS
Control
DIP2B
PWS
Angelman
SRS_11p15
BWS
SRS_UPD(7)mat
SRS_UPD(7)pat
Temple
KOS
146992500
146993000
146993500
146994000
146994500
146995000
50898000
50898500
50899000
50899500
50900000
25068750
25068760
25068770
2018000
2020000
2022000
2024000
2720000
2721000
2722000
2723000
130130000
130131000
130132000
130133000
101290400
101290600
101290800
101291000
101291200

Figure S2

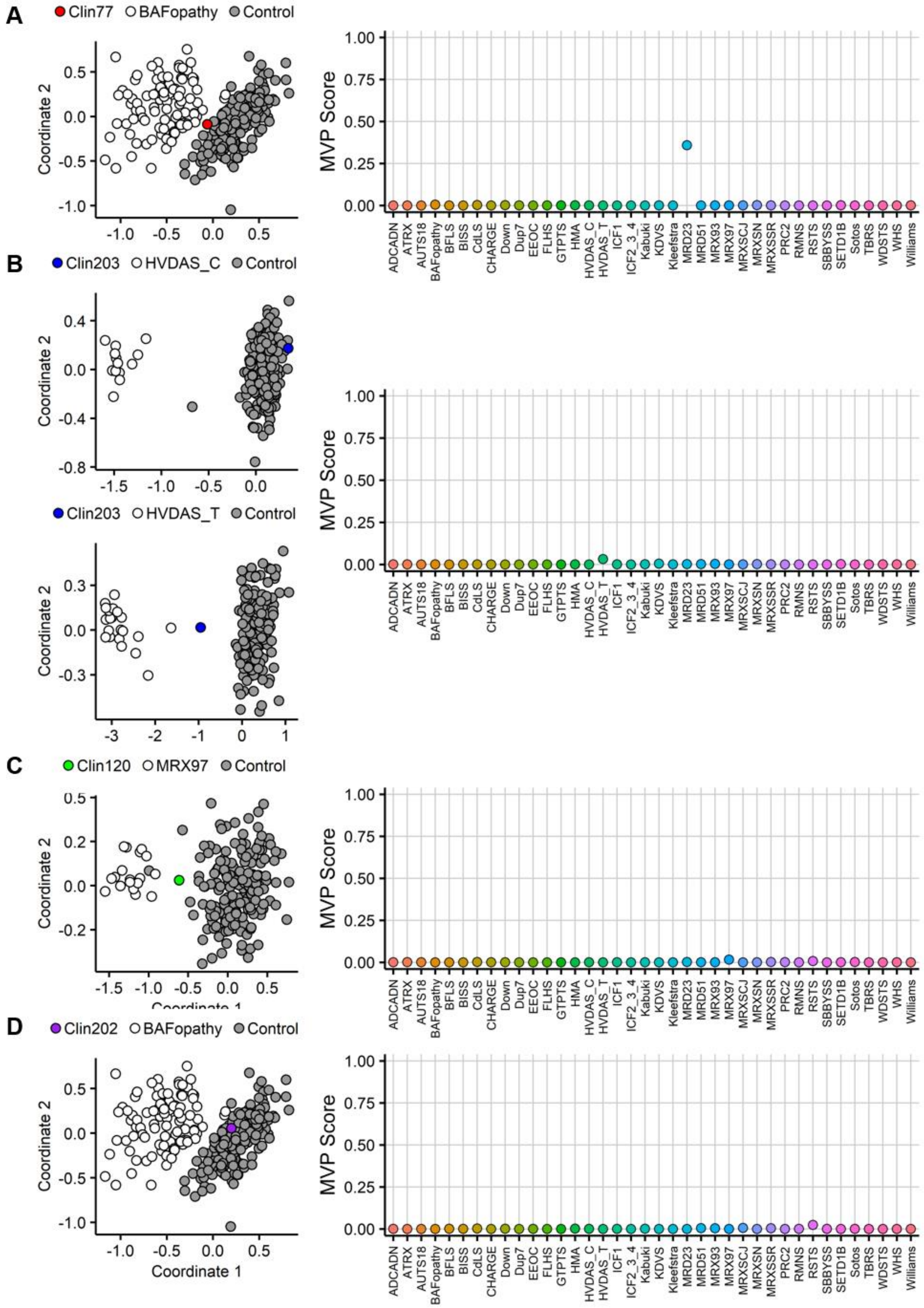
A
Clin77 BAFopathy Control
Coordinate 2
MVP Score
B
Clin203 HVDAS_C Control
Clin203 HVDAS_T Control
C
Clin120 MRX97 Control
D
Clin202 BAFopathy Control
Coordinate 1
ADCADN ATRX AUTS18 BAFopathy BFLS BISS CdLS CHARGE Down Dup7 EEOC FLHS GTPTS HMA HVDAS_C HVDAS_T ICF1 ICF2_3_4 Kabuki KDVS Kleefstra MRD23 MRD51 MRX93 MRX97 MRXSCJ MRXSN MRXSSR PRC2 RMNS RSTS SBBYSS SETD1B Sotos TBRS WDSTS WHS Williams

Table S3

Validation Cohort	Total	EpiSign Concordant
Normal Control	58	58
EpiSign Positive	123	119
Imprinting Positive	21	21
Imprinting low level mosaics (not EpiSign detectable)	9	9
Total samples	211	207

Clinical Testing Cohort	Total	EpiSign Positive	EpiSign Negative	Inconclusive
Targeted Analysis	136	48	86	2
Screening Analysis	71	9	60	2
Total Samples	207	57	146	4

Legends:

Figure S1. EpiSign DNA methylation profiles for imprinting disorders and Fragile X. Methylation levels (y-axis) are plotted against the genomic coordinates (y-axis) for subjects with the respective disorders, and control subjects. The black vertical lines indicate the borders of the region assayed for diagnostic testing, grey and black vertical lines indicate the locations of CpG probes.

Figure S2. DNA methylation (EpiSign) analysis or the inconclusive clinical cases. MDS plot and MVP score for A. Case Clin77 (ARID2:c.988_1008del, p.(Leu330_Gly336del)); B. Case Clin203 (ADNP:c.46C>G, p.(Arg16Gly); C. Case Clin120 (no genetic variant identified); and D. Case Clin202 (no genetic variant identified).
